# Pregestational body mass index, weight gain during pregnancy and perinatal outcome: a retrospective descriptive study

**DOI:** 10.31744/einstein_journal/2020AO4851

**Published:** 2019-10-28

**Authors:** Lais Assenheimer de Paula Ferreira, Carla de Azevedo Piccinato, Eduardo Cordioli, Eduardo Zlotnik

**Affiliations:** 1 Hospital Israelita Albert Einstein, São Paulo, SP, Brazil.

**Keywords:** Body mass index, Obesity, Pregnancy, Weight gain, Perinatal care, Prenatal care, Diabetes, gestational

## Abstract

**Objective:**

To analyze the pregestational body mass index and weight gain during pregnancy, and to associate data to perinatal outcomes of pregnant women from a Prenatal Care Program.

**Methods:**

A retrospective study was carried out with 151 patients seen at the Healthy Gestation Program of *Hospital Israelita Albert Einstein* . Data were collected from a medical chart review of the patients seen between March 2015 and March 2016.

**Results:**

The chance of developing gestational diabetes for obese patients in early gestation was estimated at 7.5-fold as compared to patients with low or normal body mass index.

**Conclusion:**

There was a significant association between obesity in early pregnancy and the occurrence of gestational *diabetes mellitus* in this population.

## INTRODUCTION

Prevalence of obesity in the world has practically doubled in the last 20 years.^1^ In western countries, its prevalence in pregnant women reaches 30%,^1^ and it is estimated that more than 40% of pregnant women gain weight above the recommended range for their body mass index (BMI), according to the Institute of Medicine (IOM).^2 , 3^

In 2009, the IOM issued a protocol based on clinical evidence designed to improve maternal and fetal health, by recommending a healthy maternal BMI before gestation, evaluating pregestational BMI, recommending a healthy lifestyle even in women with an adequate gestational weight, and monitoring weight gain (WG) from the beginning to the end of gestation.^2 , 4^ In this way, current evidence suggests that starting pregnancy within the healthy weight range and maintaining an adequate WG during the gestation is beneficial in the short- and long-terms for mother and child.^2 , 5^ Obese women present with an increased risk of complications in gestation, especially the hypertensive syndromes of pregnancy and gestational *diabetes mellitus* (GDM).^2 , 4^ For the neonate, there is an increased risk of macrosomia, neonatal hypoglycemia or hyperbilirubinemia, in addition to a higher risk of childhood obesity.^6 , 7^

At *Hospital Israelita Albert Einstein* (HIAE), in the city of Sao Paulo (SP), professionals from several areas have accompanied the prenatal period and the delivery, through an offer exclusively to employees and dependents, called Healthy Gestation Program (PGS - *Programa Gestação Saudável* ). The program is a part of a project, *Clínica Cuidar* [Care Clinic], which as of 2013, provides medical care to almost 28 thousand people, by conducting specialized and multidisciplinary care focused on health promotion and prevention. Knowledge of the profile and weight gain of this population of pregnant women seen by the PGS should serve as a basis for proposing new interventional measures to be applied during the prenatal or even preconception visits, improving patient care.

## OBJECTIVE

To evaluate the pregestational body mass index, weight gain during gestation, and the maternal and fetal associated complications in the population of pregnant women seen at the Healthy Gestation Program.

## METHODS

### Patients

Demographic data (age, height, number of gestations, activity at work, and marital status) from the medical records of all patients seen by the Gynecology and Obstetrics residents in PGS prenatal care, at HIAE, between March 2015 and March 2016 were analyzed. This retrospective data analysis was approved by the Research Ethics Committee (CEP) of the Research Center of *Hospital Israelita Albert Einstein* , CAAE: 59314616.4.0000.0071.

Not included were patients who presented with prior comorbidities, such as chronic arterial hypertension, and type 1 or 2 *diabetes mellitus* ; missed prenatal accompaniment; had a miscarriage; or presented with incomplete data in the medical records.

### Calculations and categories of weight gain and body mass index

For the calculation of gestational WG (GWG), the baseline or pregestational weight as reported by the patient herself during the first visit or that measured within 4 weeks of gestation. The final weight was considered as that of the last visit, a few days before the delivery. The BMI was calculated by the ratio between the weight in kilograms divided by the height in meters squared. The patients were grouped according to the four categories, as per the pregestational BMI, using as criteria low weight (BMI <18.5kg/m^2^), normal weight (BMI 18.5-24.9kg/m^2^), overweight (BMI 24.9-29.9kg/m^2^), and obesity (BMI ≥30kg/m^2^), in which the WG range during gestation was based on these pregestational BMIs.^2^

For the classification of GWG, we used as reference the pregestational BMI, as per the IOM criteria, which recommends a gain of 12.5 to 18kg for low weight women, 11.5 to 16kg for women with a normal BMI, 7 to 11.5kg in overweight women, and 5 to 9kg in the obese.^2^ The same recommendations are also adopted by the *Federação das Associações de Ginecologia e Obstetrícia* (Febrasgo) [Federation of Gynecology and Obstetrics Associations].^8^

In the “profession” category, patients were grouped in active and non-active jobs. Active jobs corresponded to those in which there was greater mobility for the woman and that demanded greater physical effort (and, therefore, a greater energy expenditure), such as nurse or licensed practicing nurses, cleaning lady, housekeeper, caregiver, kitchen worker; the less active jobs, such as switchboard operator, receptionist, secretary, were grouped into the same category.

### Statistical analyses

All statistical analyses were performed with the SAS *(* Proc MEANS and Proc FREQ) program. The association between the classification of GWG and pregestational BMI was done with the χ^2^ test. The population characteristics of patients were utilized as explanatory variables in linear or logistic regression models (Proc GLM) to understand the GWG. Additionally, the population and weight characteristics were used as explanatory variables for risk of GDM and preeclampsia in this population, in logistic regression models. The model was adjusted for potential confounding factors, and the values of the odds ratio (OR) and respective 95% confidence intervals (95%CI) were considered significant for the p value ≤0.05.

## RESULTS

**Figure 1:**
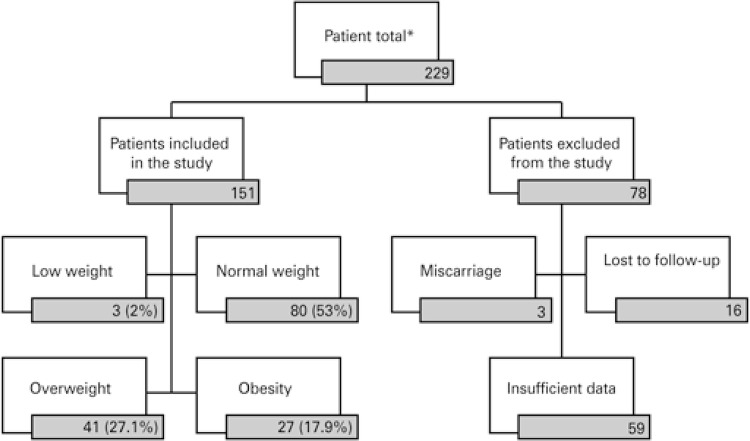
Distribution of the study patients grouped as per the pregestational body mass index

A flowchart of the inclusion of selected patients shows that, of the 229 initial patients, 151 were evaluated and grouped according to the pregestational BMI ( Figure 1 ).

Results expressed as n or n(%). * Patients with prior comorbidities were already excluded.

The mean age of patients was 30.2±5.1 years, with homogeneous distribution of occupational activity (51% active and 47.7% non-active), which did not interfere in the perinatal outcomes between the groups (p=0.7612). The mean pregestational BMI was 25.3±4.7kg/m^2^, and the mean WG during gestation was 11.4±5.4kg (p>0.05, data not presented).

Among the pregnant women with low weight in early pregnancy, 18.3% had a WG above what is recommended. Among those who were overweight, 36.6% had elevated WG, and among those with obesity, this proportion was 70.4% (p<0.001; Table 1 ).

**Table 1:** Relation between entre weight gain and initial body mass index

Classification of initial BMI	Weight gain during gestation (categories)			Total n (%)

	Low n (%)	Normal n (%)	High n (%)	
Low/Normal	31 (37.3)	37 (44.6)	15 (18.1)	83 (100)
Overweight	11 (26.8)	15 (36.6)	15 (36.6)	41 (100)
Obesity	0 (0.0)	8 (29.6)	19 (70.4)	27 (100)
Total	42 (28.0)	59 (39.3)	49 (32.7)	151 (100)

p<0.001 for the χ^2^ test of association. BMI: body mass index.

On table 2 , where the pregnant women were classified according to the pregestational BMI, the occurrence of GDM was considerably more present among the obese patients (37.03%; p=0.0069). Preeclampsia, on the other hand, did not show relevant statistical significance (p=0.5880).

**Table 2:** Population characteristics of pregnant women of the Healthy Gestation Project, classified as per the pregestational body mass index

Characteristics	Pregestational BMI pregestational				p value

	Low (n=3)	Normal (n=80)	Overweight (n=41)	Obesity (n=27)	
Initial BMI, kg/m^2^	17.6	22.3	26.5	33.4	<0.0001
Weight gain, kg	16.4	12.3	10.7	9.5	0.0319
GDM, %	0	8.7	9.7	37.0	0.0069
PE, %	0	3.7	7.3	11.1	0.5880
Weight of the NB, g	2,873.3±149.2	3,196.9±528.3	3,226.4±535.1	3,225.9±713.8	0.0942

p<0.05 was considered significant. BMI: body mass index; GDM: gestational *diabetes mellitus* ; PE: preeclampsia; NB: newborn.

When evaluating isolated factors that could contribute to the chance of GDM occurring, we noted an association with mean initial BMI (p=0.003) and obesity at early pregnancy (p=0.001; Table 3 ).

**Table 3:** Simple models for the chance of gestational *diabetes mellitus* and preeclampsia

	Odds ratio for GDM (95%CI)	p value	Odds ratio of PE (95%CI)	p value
Age, years	1.076 (0.975-1.188)	0.146	0.942 (0.830-1.069)	0.355
Number of prior gestations	1.309 (0.863-1.987)	0.205	0.527 (0.202-1.379)	0.192
Weight gain during gestation, mean (standard deviation)	0.919 (0.839-1.006)	0.066	1.051 (0.928-1.190)	0.437
Weight gain during gestation				
Low	1.004 (0.296-3.409)	0.995	4.462 (0.448-44.468)	0.202
Normal	1		1	
High	1.671 (0.573-4.874)	0.347	6.591 (0.743-58.451)	0.090
BMI Classification				
Low/normal	1		1	
Overweight	1.174 (0.323-4.263)	0.808	2.105 (0.406-10.921)	0.375
Obesity	6.387 (2.126-19.181)	0.001	3.333 (0.631-17.602)	0.156
Initial BMI	1.146 (1.046-1.256)	0.003	1.092 (0.965-1.235)	0.164

p<0.05 was considered significant.

GDM: gestational *diabetes mellitus* ; 95%CI: 95% confidence interval; PE: preeclampsia; BMI: body mass index.

Table 4 shows the effect of the joint association of all factors analyzed on the chance of occurring GDM and preeclampsia. Despite the fact that there was no evidence of interaction between the chance of occurring GDM and the majority of the variables, the association between the initial BMI classification and the occurrence of GDM, and the chance of patients with obesity at the beginning of gestation was estimated at 7.5 times the same change among patients with low or normal BMI at the start of pregnancy (p=0.005), reinforcing the simple model.

**Table 4:** Multiple model of the chance of gestational *diabetes mellitus* and preeclampsia

	Odds ratio of GDM (95%CI)	p value	Odds ratio of PE (95%CI)	p value
Weight gain during gestation				
Normal	1		1	
Low	2.044 (0.522-8.003)	0.304	5.037 (0.451-56.236)	0.189
High	1.193 (0.324-4.396)	0.791	3.910 (0.387-39.543)	0.248
Classification of BMI				
Low/normal	1		1	0.370
Overweight	0.999 (0.249-4.014)	0.999	3.146 (0.514-19.254)	0.215
Obesity	7.489 (1.819-30.828)	0.005	3.772 (0.455-31.289)	0.219
Age, years	1.087 (0.969-1.220)	0.153	0.984 (0.860-1.127)	0.817
Number of prior gestations	1.099 (0.666-1.815)	0.712	0.502 (0.183-1.375)	0.180

p<0.05 was considered significant.

GDM: gestational *diabetes mellitus* ; 95%CI: 95% confidence interval; PE: preeclampsia; BMI: body mass index.

There was no significant evidence of association between preeclampsia and any of the variables considered ( Table 4 ).

## DISCUSSION

Considering the increase in weight is a modifiable risk fact, and that its deviations can be identified and corrected during pregnancy,^9^ the promotion of healthy WG can be an important element in avoiding adverse maternal and fetal outcomes.^10^

The present study evaluated the weight gain of the population of pregnant women seen at the PGS of HIAE, as per IOM criteria,^2^ and compared the groups of pregnant women that gained adequate weight, above, and below the goals, correlating with the populational characteristics and the perinatal outcomes.

Our results indicated that in the population assessed, there are 7.5 times greater chances of GDM occurring among the patients classified as obese at early pregnancy than with the patients with low or normal BMI at the same period of pregnancy. This distinction of chance was not noted among groups with different pregestational BMIs for the occurrence of preeclampsia. Others studies evaluated weight issues and the chance of GDM or preeclampsia. In a systematic review done in 2009, Torloni et al., demonstrated the existence of a linear association of the increase in pregestational BMI with the risk of developing GDM, in which, for each 1kg/m^2^ increase in the BMI, the prevalence of GDM increased 0.92%.^11^ In comparison with normal weight women, the non-adjusted odds ratio (OR) of a low-weight woman with the development of GDM was 0.75, while the OR for overweight, moderately obese, and morbidly obese women was 1.97, 3.01, and 5.55, respectively.^11^

A study conducted with the Canadian population, in 2014, also demonstrated that women with elevated pregestational BMI were more prone to develop preeclampsia (OR: 3.5; 95%CI: 2.0-4.6 for overweight; OR: 5.3, 95%CI: 3.3-8.5 for obese), and GDM (OR: 3.0; 95%CI: 1.8-5.0 for excess weight; OR: 6.5; 95%CI: 3.7-11.2 for obese) than women with normal weight.^12^ As to hypertensive disorders of pregnancy, studies have shown that these conditions are more likely to occur in women who developed a greater GWG; however, most of these studies have significant methodological limitations, since women who developed such hypertensive comorbidities are more likely to present with edema during pregnancy than the normotensive women, and this can result in a higher GWG.^2^

Our data proved to be opposite of those published by Seabra et al., for whom the most frequent complication associated with the weight shift was preeclampsia.^13^ The GDM rate was 2.8% among those who were overweight/obese, with no association with pregestational overweight or obesity (p=0.3); on the other hand, these presented with an increased risk of developing preeclampsia (p=0.03).^13^

It is noteworthy that the mean pregestational BMI of the sample of patients studied was 25.34kg/m^2^, which corresponds to a measure of overweight.^2^ Comparing these results with other national published projects, we found that the pregestational BMI of this population was slightly superior to that found in several studies. A study carried out at the hospital of *Faculdade de Medicina de Jundiaí,* in the state of Sao Paulo, with 712 pregnant women, presented with a mean initial BMI of 24.05 (±4.74), and the total excessive WG was observed at 36.9% of pregnant women.^14^ In another retrospective study conducted in Para de Minas (State of Minas Gerais) with 64 patients between 2009 and 2011, a pregestational BMI of 23.3±4.2kg/m was found.^15^

In Brasil, according to the survey carried out by the Ministry of Health, one out of five individuals is overweight.^16^ The prevalence of obesity went from 11.8%, in 2006, to 18.9%, in 2016.^16^ These data observed for the general population in the country reflect the population of pregnant women, and many studies have observed a significant association between excess weight before the gestation and a greater frequency of excessive GWG.^17^

In the population studied, there was no association between GWG and the occurrence of preeclampsia or GDM, but other studies have shown that GWG is an important factor of pregestational events. For example, Eleutério et al., found a predominance of adequate weight gain for pregnant women with normal pregestational BMI, and excessive WG for overweight pregnant women.^15^

Thus, the pregestational BMI and the GWG are factors associated with important perinatal events and results, and therefore, identifying obese patients and those with an inadequate WG during the gestational period prove to be fundamental measures of control and prevention of maternal and fetal morbidity and mortality. Some strategies for the prevention of WG and obesity have proved to be easily executed measures and have a lower cost than treatment of already obese individuals.^9^

## CONCLUSION

The mean pregestational body mass index in the population of pregnant women evaluated corresponded to the overweight category. The pregnant women who initiated prenatal care visits in the obese category had greater difficulty in maintaining adequate weight gain control, exceeding the determinations recommended by the Institute of Medicine. There was a significant association between obesity at the beginning of pregnancy and the occurrence of gestational *diabetes mellitus* , and for greater values of body mass index, we found a greater chance of gestational *diabetes mellitus* . For this population, initiating prenatal care with a pregestational body mass index in the obesity category increased the chance of gestational *diabetes mellitus* by 7.5-fold.

## References

[1] 1. Huda SS, Brodie LE, Sattar N. Obesity in pregnancy: prevalence and metabolic consequences. Semin Fetal Neonatal Med. 2010;15(2):70-6. 10.1016/j.siny.2009.09.006 19896913

[2] 2. Institute of Medicine (US) and National Research Council (US) Committee to Reexamine IOM Pregnancy Weight Guidelines; Rasmussen KM, Yaktine AL, editors. Weight Gain During Pregnancy: Reexamining the Guidelines. Washington (DC): National Academies Press (US); 2009. 20669500

[3] 3. Gaillard R, Felix JF, Duijts L, Jaddoe VW. Childhood consequences of maternal obesity and excessive weight gain during pregnancy. Acta Obstet Gynecol Scand. 2014;93(11):1085-9. 10.1111/aogs.12506 25231923

[4] 4. Rasmussen KM, Abrams B, Bodnar LM, Butte NF, Catalano PM, Maria Siega-Riz A. Recommendations for weight gain during pregnancy in the context of the obesity epidemic. Obstet Gynecol. 2010;116(5):1191-5. Review. 10.1097/AOG.0b013e3181f60da7 PMC4288953 20966705

[5] 5. Davies GA, Maxwell C, McLeod L, Gagnon R, Basso M, Bos H, Delisle MF, Farine D, Hudon L, Menticoglou S, Mundle W, Murphy-Kaulbeck L, Ouellet A, Pressey T, Roggensack A, Leduc D, Ballerman C, Biringer A, Duperron L, Jones D, Lee LS, Shepherd D, Wilson K; Society of Obstetricians and Gynaecologists of Canada. SOGC Clinical Practice Guidelines: Obesity in pregnancy. No. 239, February 2010. Int J Gynaecol Obstet. 2010;110(2):167-73. 10.1016/j.ijgo.2010.03.008 20641146

[6] 6. Hedderson MM, Weiss NS, Sacks DA, Pettitt DJ, Selby JV, Quesenberry CP, et al. Pregnancy weight gain and risk of neonatal complications: macrosomia, hypoglycemia, and hyperbilirubinemia. Obstet Gynecol. 2006;108(5):1153-61. 10.1097/01.AOG.0000242568.75785.68 17077237

[7] 7. Oken E, Taveras EM, Kleinman KP, Rich-Edwards JW, Gillman MW. Gestational weight gain and child adiposity at age 3 years. Am J Obstet Gynecol. 2007; 196(4):322. e1-8. 10.1016/j.ajog.2006.11.027 PMC1899090 17403405

[8] 8. Federação Brasileira das Associações de Ginecologia e Obstetrícia (FEBRASGO). Manual de Assistência Pré-Natal [Internet]. São Paulo: FEBRASGO; 2014 [citado 2019 Jun 14]. Disponível em: http://www.abenforj.com.br/site/arquivos/manuais/304_Manual_Pre_natal_25SET.pdf

[9] 9. Tanentsapf I, Heitmann BL, Adegboye AR. Systematic review of clinical trials on dietary interventions to prevent excessive weight gain during pregnancy among normal weight, overweight and obese women. BMC Pregnancy Childbirth. 2011;11(1):81. Review. 10.1186/1471-2393-11-81 PMC3215955 22029725

[10] 10. Marmitt LP, Gonçalves CV, Cesar JA. Healthy gestational weight gain prevalence and associated risk factors: A population-based study in the far South of Brazil. Rev Nutr. 2016;29(4):445-55.

[11] 11. Torloni MR, Betrán AP, Horta BL, Nakamura MU, Atallah AN, Moron AF, et al. Prepregnancy BMI and the risk of gestational diabetes: a systematic review of the literature with meta-analysis. Obes Rev. 2009;10(2):194-203. Review. 10.1111/j.1467-789X.2008.00541.x 19055539

[12] 12. Vinturache A, Moledina N, McDonald S, Slater D, Tough S. Pre-pregnancy Body Mass Index (BMI) and delivery outcomes in a Canadian population. BMC Pregnancy Childbirth. 2014;14(1):422. 10.1186/s12884-014-0422-y PMC4300169 25528667

[13] 13. Seabra G, Padilha PC, de Queiroz JA, Saunders C. Sobrepeso e obesidade pré-gestacionais: prevalência e desfechos associados à gestação. Rev Bras Ginecol Obstet. 2011;33(11):348-53. 22267113

[14] 14. da Fonseca MR, Laurenti R, Marin CR, Traldi MC. Ganho de peso gestacional e peso ao nascer do concepto: estudo transversal na região de Jundiaí, São Paulo, Brasil. Cien Saude Colet. 2014;19(5):1401-7. 24897205

[15] 15. Eleutério BM, Araújo GL, Silveira LP, Anastácio LR. Perfil nutricional materno e estado nutricional neonatal, na cidade de Pará de Minas - MG. Rev Med Minas Gerais. 2013;23(3):311-7.

[16] 16. Brasil. Governo do Brasil. Doença crônica: Obesidade cresce 60% em dez anos no Brasil [Internet]. Brasília: Governo do Brasil; 2017 [citado 2018 Dez 17]. Disponível em: http://www.brasil.gov.br/saude/2017/04/obesidade-cresce-60-em-dez-anos-no-brasil

[17] 17. Magalhães EI, Maia DS, Bonfim CF, Netto MP, Lamounier JA, Rocha DS. Prevalência e fatores associados ao ganho de peso gestacional excessivo em unidades de saúde do sudoeste da Bahia. Rev Bras Epidemiol. 2015; 18(4):858-69. 10.1590/1980-5497201500040014 26982300

