# Supplementary material for: Pregestational body mass index, weight gain during pregnancy and perinatal outcome: a retrospective descriptive study
Source: Einstein (Sao Paulo). 2019 Oct 28;18:eAO4851. doi: 10.31744/einstein_journal/2020AO4851 (PMC6896599; doi:10.31744/einstein_journal/2020AO4851)
Supplement: Translation

# Translation: Índice de massa corporal pré-gestacional, ganho de peso na gestação e resultado perinatal: estudo descritivo retrospectivo

**Authors:** Lais Assenheimer de Paula Ferreira, Carla de Azevedo Piccinato, Eduardo Cordioli, Eduardo Zlotnik

**Affiliations:** 1 Hospital Israelita Albert Einstein, São Paulo, SP, Brasil.

**Keywords:** Índice de massa corporal, Obesidade, Gestação, Ganho de peso, Assistência perinatal, Cuidado pré-natal, Diabetes gestacional

## Abstract

**Objetivo:**

Analisar o índice de massa corporal pré-gestacional e o ganho de peso na gestação, e associar os dados a desfechos perinatais de gestantes de um Programa de Gestação Saudável.

**Métodos:**

Estudo retrospectivo realizado com 151 pacientes atendidas no Programa de Gestação Saudável do Hospital Israelita Albert Einstein. Os dados foram coletados a partir da revisão de prontuário das pacientes atendidas entre março de 2015 e março de 2016.

**Resultados:**

A chance de desenvolvimento de diabetes gestacional para as pacientes com obesidade no início da gestação foi estimada em 7,5 vezes a mesma chance entre as pacientes com índice de massa corporal baixo ou normal no início da gravidez.

**Conclusão:**

Houve associação significativa entre obesidade no início da gravidez e a ocorrência de *diabetes mellitus* gestacional nesta população.

## INTRODUÇÃO

A prevalência de obesidade no mundo praticamente dobrou nos últimos 20 anos.^1^ Nos países ocidentais, sua prevalência em gestantes chega a 30%,^1^ e estima-se que mais de 40% das gestantes ganhem peso acima da faixa recomendada para seu índice de massa corporal (IMC), segundo o *Institute of Medicine* (IOM).^2 , 3^

Em 2009, o IOM emitiu um protocolo baseado em evidências clínicas com o objetivo de melhorar a saúde materna e fetal, recomendando IMC materno saudável antes da gestação, avaliando o IMC pré-gestacional, aconselhando um estilo de vida saudável mesmo em mulheres com peso gestacional adequado e monitorando ganho de peso (GP) do começo ao final da gestação.^2 , 4^ Desta forma, evidências atuais sugerem que começar a gestação dentro da faixa de peso saudável e manter um adequado GP durante a gestação é benéfico para mãe e criança, a curto e longo prazo.^2 , 5^ Mulheres obesas apresentam risco aumentado de complicações na gestação, incluindo principalmente as síndromes hipertensivas da gestação e *diabetes mellitus* gestacional (DMG).^2 , 4^ Para o neonato, há risco aumentado de macrossomia, hipoglicemia ou hiperbilirrubinemia neonatal, além de haver maior risco de obesidade infantil.^6 , 7^

No Hospital Israelita Albert Einstein (HIAE), em São Paulo (SP), profissionais de diversas áreas têm acompanhamento de pré-natal e do parto por um programa oferecido exclusivamente a colaboradores e dependentes, chamado Programa Gestação Saudável (PGS). O PGS faz parte do projeto Clínica Cuidar, que, desde 2013, disponibiliza assistência médica para quase 28 mil pessoas, realizando atendimento especializado e multidisciplinar, com foco na promoção e na prevenção da saúde. O conhecimento do perfil e do ganho ponderal desta população de gestantes atendida pelo PGS deve servir de base para propor novas medidas de intervenção a serem aplicadas durante as consultas de pré-natal ou até pré-concepcional, melhorando a assistência às pacientes.

## OBJETIVO

Avaliar o índice de massa corporal pré-gestacional, o ganho de peso durante a gestação, e as intercorrências maternas e fetais associadas na população de gestantes atendidas no Programa Gestação Saudável.

## MÉTODOS

### Pacientes

Foram analisados os dados demográficos (idade, altura, número de gestações, atividade no trabalho e estado civil) de prontuários de todas as pacientes atendidas pelos residentes de Ginecologia e Obstetrícia, no pré-natal do PGS do HIAE, entre março de 2015 a março de 2016. Esta análise retrospectiva dos dados foi aprovada pela Comissão de Ética em Pesquisa (CEP) do Centro de Pesquisas do Hospital Israelita Albert Einstein, CAAE: 59314616.4.0000.0071.

Não foram incluídas no estudo as pacientes que apresentavam comorbidades prévias como, por exemplo, hipertensão arterial crônica e *diabetes mellitus* tipos 1 ou 2; perderam acompanhamento no pré-natal; apresentaram abortamento; ou apresentavam dados incompletos no prontuário para o estudo.

### Cálculos e categorias de ganho de peso e índice de massa corporal

Para o cálculo do GP na gestação (GPG), foi considerado o peso inicial ou pré- gestacional como o referido pela própria paciente na primeira consulta ou aferido até 4 semanas de gestação. O peso final foi considerado como o da última consulta, poucos dias antes do parto. Foi calculado o IMC, pela razão entre o peso em quilograma dividido pela altura em metros ao quadrado. As pacientes foram agrupadas de acordo com quatro categorias, segundo o IMC pré- gestacional, utilizando como critérios baixo peso (IMC <18,5kg/m^2^), peso normal (IMC 18,5-24,9kg/m^2^), sobrepeso (IMC 24,9-29,9kg/m^2^) e obesidade (IMC ≥30kg/m^2^), sendo a faixa de GP durante a gestação baseada nestes IMCs pré-gestacionais.^2^

Para classificação do GPG, utilizamos como referência o IMC pré-gestacional, segundo os critérios do IOM, que recomenda ganho 12,5 a 18kg para mulheres com baixo peso, 11,5 a 16kg em mulheres com IMC normal, 7 a 11,5kg nas mulheres com sobrepeso, e 5 a 9kg nas obesas.^2^ As mesmas recomendações também são adotadas pela Federação das Associações de Ginecologia e Obstetrícia (Febrasgo).^8^

Na categoria “profissão”, as pacientes foram agrupadas em cargos ativos e não ativos. Cargos ativos corresponderam àqueles em que havia maior mobilidade da mulher e demandasse maior esforço físico (e, portanto, maior gasto energético), como enfermeira e auxiliar de enfermagem, faxineira, camareira, cuidadora, copeira; os cargos menos ativos, como telefonista, atendente, secretária, foram agrupados em uma mesma categoria.

### Análises estatísticas

Todas as análises estatísticas foram realizadas no programa SAS *(* Proc MEANS e Proc FREQ). A associação entre classificação de GPG e IMC pré- gestacional foi feita pelo teste do χ^2^. As características populacionais das pacientes foram utilizadas como variáveis explicativas em modelos de regressão linear ou logística (Proc GLM) para entender o GPG. Além disso, as características populacionais e ponderais foram utilizadas como variáveis explicativas para o risco de DMG e pré-eclâmpsia nesta população, em modelos de regressão logística. O modelo foi ajustado para potenciais fatores de confusão, e os valores de razão de chance (RC) e respectivos intervalos de confiança de 95% (IC95%) foram considerados significantes para o valor de p≤0,05.

## RESULTADOS

Um fluxograma de inclusão de pacientes selecionadas mostra que, dos 229 pacientes iniciais, foram avaliados 151 pacientes e agrupadas de acordo com o IMC pré-gestacional ( Figura 1 ).

**Figura 1:** Distribuição das pacientes do estudo agrupadas de acordo com o IMC pré-gestacional

Resultados expressos em n ou n (%). *Já excluídas as pacientes com comorbidades prévias.

A média de idade das pacientes foi de 30,2±5,1 anos, com distribuição homogênea da atividade ocupacional (51% ativa e 47,7% não ativa), o que não interferiu nos desfechos perinatais entre os grupos (p=0,7612). O IMC pré- gestacional médio foi 25,3±4,7kg/m^2^e o GP médio durante a gestação foi de 11,4±5,4kg (p>0,05, dados não apresentados).

Dentre as gestantes com baixo peso no início da gravidez, 18,3% tiveram GP acima do recomendado. Entre aquelas com sobrepeso, 36,6% tiveram GP elevado e entre as com obesidade, esta proporção foi de 70,4% (p<0,001; tabela 1 ).

**Tabela 1:** Relação entre ganho de peso e índice de massa corporal inicial

Classificação de IMC inicial	Ganho de peso durante a gestação (categorias)			Total n (%)

	Baixo n (%)	Normal n (%)	Alto n (%)	
Baixo/Normal	31 (37,3)	37 (44,6)	15 (18,1)	83 (100)
Sobrepeso	11 (26,8)	15 (36,6)	15 (36,6)	41 (100)
Obesidade	0 (0,0)	8 (29,6)	19 (70,4)	27 (100)
Total	42 (28,0)	59 (39,3)	49 (32,7)	151 (100)

Valor de p para o teste de associação de χ^2^: <0,001. IMC: índice de massa corporal.

Na tabela 2 , em que as gestantes foram classificadas de acordo com o IMC pré-gestacional, a ocorrência de DMG esteve consideravelmente mais presente dentre as pacientes obesas (37,03%; p=0,0069). Já a pré-eclâmpsia não apresentou significância estatística relevante (p=0,5880).

**Tabela 2:** Características populacionais das gestantes do Projeto Gestação Saudável, classificadas de acordo com o índice de massa corporal pré-gestacional

Características	IMC pré-gestacional				Valor de p

	Baixo (n=3)	Normal (n=80)	Sobrepeso (n=41)	Obesidade (n=27)	
IMC inicial, kg/m^2^	17,6	22,3	26,5	33,4	<0,0001
Ganho de peso, kg	16,4	12,3	10,7	9,5	0,0319
DMG, %	0	8,7	9,7	37,0	0,0069
PE, %	0	3,7	7,3	11,1	0,5880
Peso RN, g	2.873,3±149,2	3.196,9±528,3	3.226,4±535,1	3.225,9±713,8	0,0942

Valor de p considerado significativo <0,05. IMC: índice de massa corporal; DMG: *diabetes mellitus* gestacional; PE: pré-eclâmpsia; RN: recém-nascido.

Ao se avaliarem isoladamente fatores que podiam contribuir para a chance de ocorrer DMG, observamos associação com IMC inicial médio (p=0,003) e obesidade no início da gravidez (p= 0,001; Tabela 3 ).

**Tabela 3:** Modelos simples para a chance de *diabetes mellitus* g *e* stacional e pré-eclâmpsia

	Razão de chances de DMG (IC95%)	Valor de p	Razão de chances de PE (IC95%)	Valor de p
Idade, anos	1,076 (0,975-1,188)	0,146	0,942 (0,830-1,069)	0,355
Número de gestações anteriores	1,309 (0,863-1,987)	0,205	0,527 (0,202-1,379)	0,192
Ganho de peso durante a gestação, média (desvio padrão)	0,919 (0,839-1,006)	0,066	1,051 (0,928-1,190)	0,437
Ganho de peso durante a gestação				
Baixo	1,004 (0,296-3,409)	0,995	4,462 (0,448-44,468)	0,202
Normal	1		1	
Alto	1,671 (0,573-4,874)	0,347	6,591 (0,743-58,451)	0,090
Classificação de IMC				
Baixo/normal	1		1	
Sobrepeso	1,174 (0,323-4,263)	0,808	2,105 (0,406-10,921)	0,375
Obesidade	6,387 (2,126-19,181)	0,001	3,333 (0,631-17,602)	0,156
IMC inicial	1,146 (1,046-1,256)	0,003	1,092 (0,965-1,235)	0,164

Valor de p considerado significativo <0,05.

DMG: *diabetes mellitus* gestacional; IC95%: intervalo de confiança de 95%; PE: pré-eclâmpsia; IMC: índice de massa corporal.

A tabela 4 mostra o efeito da associação conjunta de todos os fatores analisados na chance de ocorrência de DMG e pré-eclâmpsia. Apesar de não terem ocorrido evidências de interação entre a chance de ocorrer DMG e a maioria das variáveis, foi clara a associação entre classificação de IMC inicial e ocorrência de DMG, e a chance de as pacientes com obesidade no início da gestação foi estimada em 7,5 vezes a mesma chance entre as pacientes com IMC baixo ou normal no início da gravidez (p=0,005), reforçando o resultado do modelo simples.

**Tabela 4:** Modelo múltiplo para a chance de *diabetes mellitus* gestacional e pré-eclâmpsia

	Razão de chances de DMG (IC95%)	Valor de p	Razão de chances de PE (IC95%)	Valor de p
Ganho de peso na gestação				
Normal	1		1	
Baixo	2,044 (0,522-8,003)	0,304	5,037 (0,451-56,236)	0,189
Alto	1,193 (0,324-4,396)	0,791	3,910 (0,387-39,543)	0,248
Classificação de IMC				
Baixo/normal	1		1	0,370
Sobrepeso	0,999 (0,249-4,014)	0,999	3,146 (0,514-19,254)	0,215
Obesidade	7,489 (1,819-30,828)	0,005	3,772 (0,455-31,289)	0,219
Idade, anos	1,087 (0,969-1,220)	0,153	0,984 (0,860-1,127)	0,817
Número de gestações anteriores	1,099 (0,666-1,815)	0,712	0,502 (0,183-1,375)	0,180

Valor de p considerado significativo <0,05.

DMG: *diabetes mellitus* gestacional; IC95%: intervalo de confiança de 95%; PE: pré-eclâmpsia; IMC: índice de massa corporal.

Não houve evidência significativa de associação da pré-eclâmpsia com nenhuma das variáveis consideradas ( Tabela 4 ).

## DISCUSSÃO

Considerando que o aumento de peso é um fator de risco modificável, e seus desvios podem ser identificados e corrigidos durante a gravidez,^9^ a promoção do GP saudável pode ser elemento importante para evitar desfechos maternos e fetais adversos.^10^

O presente estudo avaliou o ganho ponderal da população de gestantes atendidas no PGS do HIAE, segundo critérios do IOM,^2^ e comparou os grupos de gestantes que ganharam peso adequado, acima e abaixo das metas, correlacionando com as características populacionais e os desfechos perinatais.

Nossos resultados indicam que, na população avaliada, há 7,5 vezes maior chance de ocorrer DMG dentre as pacientes classificadas como obesas no início da gestação do que com as pacientes com IMC baixo ou normal no início da gravidez. Não houve esta distinção de chance entre grupos com diferentes IMC pré-gestacionais para ocorrência de pré-eclâmpsia. Outros estudos avaliaram questões ponderais e chance de DMG ou pré-eclâmpsia. Torloni et al., em revisão sistemática realizada em 2009, demonstraram a existência de associação linear do aumento do IMC pré-gestacional com risco de desenvolvimento de DMG, sendo que, para cada aumento de 1kg/m^2^no IMC, a prevalência de DMG aumentou 0,92%.^11^ Em comparação com mulheres com peso normal, a razão de chance (RC) não ajustada de uma mulher com baixo peso em desenvolvimento de DMG foi de 0,75, enquanto a RC para mulheres com sobrepeso, moderadamente obesas e com obesidade mórbida foi de 1,97, 3,01 e 5,55, respectivamente.^11^

Um estudo realizado com população canadense, em 2014, também demonstrou que mulheres com IMC pré-gestacional elevado foram mais propensas a desenvolver pré-eclâmpsia (RC: 3,5; IC95%: 2,0-4,6 para sobrepeso; RC: 5,3, IC95%: 3,3-8,5 para obesos) e DMG (RC: 3,0; IC95%: 1,8-5,0 para excesso de peso; RC: 6,5; IC95%: 3,7-11,2 para obesos) do que mulheres com peso normal.^12^ Sobre as desordens hipertensivas na gestação, os estudos têm demonstrado que estas estão mais propensas a acontecer nas mulheres que desenvolveram maior GPG; entretanto, a maioria desses estudos tem importantes limitações metodológicas, pois as mulheres que desenvolvem tais comorbidades hipertensivas são mais propensas a apresentarem edema durante a gestação do que as normotensas, e isso, por sua vez, pode resultar em maior GPG.^2^

Nossos dados mostraram-se opostos aos publicados no estudo de Seabra et al., para quem a intercorrência mais frequente associada ao desvio ponderal estudado foi a pré-eclâmpsia.^13^ A taxa de DMG foi de 2,8% entre aquelas com sobrepeso/obesidade, não estando associada com sobrepeso ou obesidade pré-gestacional (p=0,3); em contrapartida, estas apresentaram risco aumentado de desenvolver pré-eclâmpsia (p=0,03).^13^

Há de se destacar que o IMC médio pré-gestacional da amostra de pacientes estudada foi de 25,34 kg/m^2^, o que corresponde a uma medida de sobrepeso.^2^ Comparando estes resultados com outros trabalhos nacionais publicados, encontramos que o IMC pré-gestacional desta população esteve ligeiramente superior ao encontrado em diversos estudos. Estudo realizado no hospital da Faculdade de Medicina de Jundiaí, em São Paulo, com 712 gestantes apresentou média do IMC inicial de 24,05 (±4,74), e o GP total excessivo foi observado em 36,9% das gestantes.^14^ Em outro estudo retrospectivo desenvolvido em Pará de Minas (MG) com 64 pacientes entre 2009 e 2011, foi encontrado IMC pré-gestacional de 23,3±4,2kg/m.^15^

No Brasil, segundo levantamento do Ministério da Saúde, uma em cada cinco pessoas está acima do peso.^16^ A prevalência da obesidade passou de 11,8%, em 2006, para 18,9%, em 2016.^16^ Estes dados observados para a população geral no país refletem na população de gestantes, e muitos trabalhos observaram associação significativa entre excesso de peso antes da gestação e maior frequência de GPG excessivo.^17^

Na população estudada não houve associação entre GPG e ocorrência de pré-clâmpsia ou DMG, porém outros estudos mostram que GPG é importante fator de eventos pré-gestacionais. Por exemplo, Eleutério et al., encontraram predominância de ganho ponderal adequado para gestantes com IMC pré- gestacional normal e excessivo para gestantes com sobrepeso.^15^

Logo, o IMC pré-gestacional e o GPG são fatores associados a importantes eventos e resultados perinatais, e, portanto, identificar as pacientes obesas e com GP inadequado no período gestacional revela-se fundamental medida de controle e prevenção de morbimortalidade materno-fetal. Algumas estratégias para prevenção de GP e obesidade têm provado serem medidas de fácil execução e têm menor custo do que o tratamento de pessoas já obesas.^9^

## CONCLUSÃO

O índice de massa corporal pré-gestacional médio da população de gestantes avaliadas correspondeu à categoria de sobrepeso. As gestantes que iniciaram o pré-natal na categoria obesidade tiveram maior dificuldade em manter o controle de ganho de peso adequado, ultrapassando as determinações preconizadas pelo *Institute of Medicine* . Houve associação significativa entre obesidade no início da gravidez e ocorrência de *diabetes mellitus* gestacional, e para maiores valores de índice de massa corporal encontramos maior chance de *diabetes mellitus* gestacional. Para esta população, iniciar o pré-natal com índice de massa corporal pré-gestacional na categoria obesidade aumentou em 7,5 vezes a chance de ocorrência de *diabetes mellitus* gestacional.
